# Association between polyunsaturated fatty acids dietary intake and pulmonary function among American children: NHANES 2007–2012

**DOI:** 10.3389/fnut.2025.1573140

**Published:** 2025-05-21

**Authors:** Mengmeng Ding, Shuyan Qie, Hanming Wang

**Affiliations:** Rehabilitation Treatment Center, Beijing Rehabilitation Hospital, Affiliated to Capital Medical University, Beijing, China

**Keywords:** children, pulmonary function, polyunsaturated fatty acids, dietary intake, NHANES

## Abstract

**Background:**

The existing evidence regarding the protective effect of polyunsaturated fatty acids (PUFAs) on pulmonary function remains a subject of considerable controversy. Based on this, we further investigated the correlation of PUFAs intake in diet with pulmonary function in healthy American children.

**Methods:**

A cross-sectional survey was conducted using the National Health and Nutrition Examination Survey (NHANES) database of children aged 6–17 in the United States from 2007 to 2012. The correlation of PUFAs intake in diet with pulmonary function was investigated through weighted multivariate linear regression and restricted cubic spline (RCS) curve visualization analysis. Subgroup analysis was carried out to further investigate the robustness of the results and potential interactions in terms of gender, race, age of child, age of mother at birth, and poverty-income ratio (PIR).

**Results:**

Altogether 2,508 participants were enrolled in this research. After adjusting for confounders, PUFAs intake was positively correlated with forced expiratory volume in 1 s (FEV1) (*β* = 7.525; 95%CI: 2.935–12.12; *p* = 0.002) and FVC (*β* = 9.138; 95%CI: 4.389, 13.89; *P* < 0.001). The modeling of PUFAs subtypes revealed that FEV1 and FVC increased with increasing intake of omega-3 and omega-6 (*p* < 0.01). The RCS results showed a non-linear relationship (*p* < 0.001) of PUFAs and omega-6 with FEV1 and FVC. A subgroup analysis in this research revealed an interaction of PUFAs intake with the gender of children, with PUFAs having a better protective effect on pulmonary function in males than in females (FEV1: *p* = 0.017; FVC: *p* = 0.022).

**Conclusion:**

The total intake of PUFAs in the diet was positively correlated with pulmonary function in children in the United States, and this correlation was more significant in the male population. The results of this study further confirmed that dietary supplementation of PUFAs was beneficial for improving pulmonary function in children.

## 1 Introduction

With the increasing severity of air pollution, respiratory health has gradually becoming a research hotspot. Pulmonary function is an important indicator to assess the health of the respiratory system, and also a predictive index for the incidence and mortality of cardiopulmonary diseases, especially in children (1). The growth and development of lungs during childhood plays a vital role in determining pulmonary function during adulthood (2). Increasing evidence has suggested that pulmonary dysfunction formed in childhood may persist into adulthood (1, 3), and early life pulmonary dysfunction is correlated with premature onset of chronic obstructive pulmonary disease (COPD) and other chronic diseases in adulthood (4, 5). Furthermore, pulmonary dysfunction is also an important cause of childhood mortality, and low level of FEV1 is a sign of premature death in children from all causes (6).

Children’s pulmonary function development is influenced by multiple factors, including their living environment, lifestyle, and genetics (7). Dietary factor, as an important factor in lifestyle, can regulate the impact of adverse environmental exposure or genetic susceptibility on the lungs, playing an important role in pulmonary function protection and respiratory health (8). Polyunsaturated fatty acids (PUFAs) are fatty acids that contain two or more double bonds and can be further classified into two groups, omega-3 and omega-6. Most of them cannot be synthesized in the body and must be obtained from the diet. They are essential fatty acids that are crucial for maintaining normal physiological functions (9, 10).

Omega-3 polyunsaturated fatty acids (PUFAs) include *α*-linolenic acid (ALA) and its long-chain derivatives stearidonic acid (SDA), eicosapentaenoic acid (EPA), docosapentaenoic acid (DPA), and docosahexaenoic acid (DHA) (11). Based on previous research findings, omega-3 has anti-inflammatory properties that can regulate human immune function to reduce inflammation and improve pulmonary function (12–16). In addition, it can also reduce lung damage caused by air pollution (17, 18). However, there is still significant controversy regarding the effectiveness of omega-3 fatty acids in improving pulmonary function, with some studies yielding contradictory results, which suggests no correlation between pulmonary function and the intake of omega-3 fatty acid (19–25). The differences in research findings have led to significant uncertainty in the correlation between the intake of omega-3 and pulmonary function. In addition, existing studies on the correlation between the intake of omega-3 and pulmonary function mainly focus on adults, which leads to greater uncertainty in the correlation between the intake of omega-3 fatty acid and pulmonary function in children compared to adults.

Omega-6 fatty acids include linoleic acid (LA), gamma linolenic acid (GLA), conjugated linoleic acid (CLA), and arachidonic acid (AA) (26). Unlike the pro-inflammatory properties of omega-3, excessive intake of omega-6 fatty acids may lead to an increased inflammatory response, while appropriate intake of omega-6 fatty acids is necessary for maintaining physical health (26). Omega-6 fatty acids are one of the sources of bioactive molecules in the lungs, providing a biological basis for respiration (27). Although these correlations have been observed, no studies directly exploring the impact of omega-6 on pulmonary function have been conducted.

Given the controversy and uncertainty regarding the relationship between dietary intake of PUFAs and pulmonary function in children, we have evaluated the association between dietary intake of PUFAs and pulmonary function in a sample of American children aged 6–17 years by integrating the NHANES database from 2007 to 2012 from three aspects: PUFAs, omega-3, and omega-6.

## 2 Methods

### 2.1 Study population

A complex stratified, multistage sampling strategy was applied in NHANES database, which is a population-based nationwide cross-sectional survey. The main target population was non-institutional civilians living in the United States, who underwent laboratory evaluations related to nutrition and health, physical examinations, and questionnaire surveys. The Ethics Review Committee of the National Center for Health Statistics approved actions in NHANES, and each participant provided written informed consent.

The data from NHANES database from 2007 to 2012 were included in this study, and only participants aged 6 and above underwent lung capacity testing. Therefore, children aged 6–17 years were included in this study, with exclusion criteria including children beyond the age range, articles with missing data, and data with quality levels of C, D, and F.

### 2.2 Evaluation of PUFAs intake in diet

PUFAs intake data was based on NHANES dietary questionnaire. The total PUFAs intake in diet was evaluated through two 24-h dietary recall interviews. The intake of PUFAs was represented by the average of two interviews. The first interview was performed face-to-face at a mobile examination center (MEC), and the second interview was carried out through follow-up calls approximately 3–10 days later. A detailed description of the interview procedure could be found in the Diet Interview Section on the website of NHANES^1^. In this study, PUFAs included omega-3 PUFAs and omega-6 PUFAs. Among them, omega-3 PUFAs included *α*-linolenic acid (gm, ALA), stearidonic acid (gm, SDA), eicosapentaenoic acid (gm, EPA), docosapentaenoic acid (gm, DPA), docosahexaenoic acid (gm, DHA). Omega−6 PUFAs included linoleic acid (gm, LA) and arachidonic acid (gm, AA).

### 2.3 Measurement of pulmonary function outcome

Participants aged 6–17 years underwent spirometry, but those who had chest pain, difficulty breathing, were currently undergoing thoracic, abdominal, or eye surgery, have recently suffered a stroke or heart attack, had a history of tuberculosis or hemoptysis were not tested. The predicted lung function (FEV1, FVC) was tested based on the NHANES III equation (28). To ensure the accuracy of the data, only pulmonary function test data with quality levels A (exceeding the Data Collection Standards of the American Thoracic Society) and B (complying with the Data Collection Standards of the American Thoracic Society) were adopted.

### 2.4 Covariates

Based on previous research, the following potential confounders that may affect children’s pulmonary function outcomes were selected (29–36). Categorical variables included gender, race, family income to poverty ratio, and whether the mother smoked during pregnancy. Continuous variables included age, serum cotinine, body Mass Index (BMI), age of mother at birth, child’s birth weight. Race was classified as Mexican American, non-Hispanic White, non-Hispanic Black, and other races. PIR standard involved low-income (PIR less than or equal to 1.3), middle-income (PIR more than 1.31 and less than or equal to 3.5), and high-income (PIR more than 3.5), whether the mother smoked during pregnancy (yes or no).

### 2.5 Statistical analysis

Continuous variables conforming to a normal distribution were represented as mean ± standard deviation (SD), while variables with a skewed distribution were represented as median and interquartile range (IQR). Categorical variables were presented as frequencies and percentages. One-way ANOVA, Kruskal–Wallis test, and Chi-square test were adopted to compare between-group differences in continuous and categorical variables. Furthermore, three weighted linear regression models were constructed to explore the correlation of PUFAs and its subtypes omega-3 and omega-6 with pediatric pulmonary function in multivariate regression analysis. Model 1 was not adjusted, Model 2 was adjusted based on gender, age, race and BMI. Model 3 was adjusted for PIR, cotinine, whether the mother smoked during pregnancy, age of mother at birth, child’s birth weight based on Model 2. Finally, the non-linear correlation of dietary PUFAs, omega-3, and omega-6 exposure with pulmonary function was estimated using the RCS. The interaction of PUFAs, omega-3, and omega-6 in diet with stratified covariates was explored using a likelihood ratio test. Meanwhile, subgroup analysis was carried out to estimate whether gender, race, PIR, and BMI altered the impact of omega-3 on pulmonary function in children. R software (version 4.1.2) was adopted to perform statistical analyses. Differences achieving *p* < 0.05 were considered statistically significant.

## 3 Results

### 3.1 Baseline characteristics of participants

**Figure 1:**
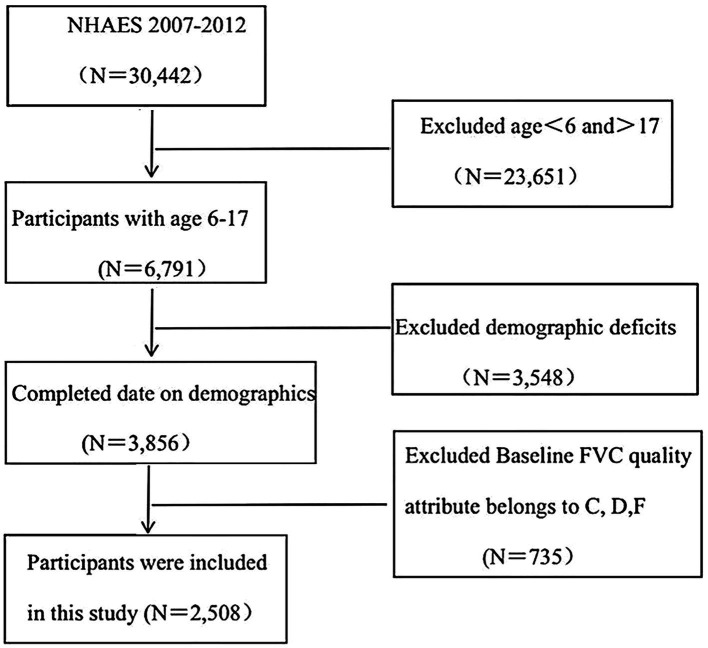
Flowchart for data screening.

After excluding individuals with age not meeting the requirements (*n* = 23,651), missing data (*n* = 3,548), and baseline FVC quality attributes classified as C, D, and F (*n* = 735), a total of 2,508 subjects were included (Figure 1). According to the tertiles of total PUFAs intake, the children subjects were divided into three groups, Q1, Q2, and Q3 to describe the baseline characteristics, with a total of 894 individuals in Q1, 816 in Q2, and 798 in Q3. Among the 2,508 participants, 49% were male, the majority of mothers did not smoke during pregnancy (88%), and 60% of the participants had children aged ≤11 years. The low-income group accounted for 42%, and the high-income group accounted for 22%. The total FEV1 and FVC of the subjects were 2,367 (1,789, 3,064; *p* = 0.057) and 2,786 (2,092, 3,518; *p* = 0.032), respectively. The grouping results based on tertiles showed that the FVC value of the Q3 group was significantly higher than that of the Q1 and Q2 groups (*p* < 0.05) (Table 1).

**Table 1:** Characteristics of participants included in the study from NHANES 2007–2012.

Characteristic	Overall	Q1^4^	Q2^4^	Q3^4^	*p*-value^3^
	*N* = 2,508^1^	*N* = 894^1^	*N* = 816^1^	*N* = 798^1^	
Gender (%)^2^					0.01
Male	1,266(49%)	402(44%)	406(47%)	458(56%)	
Female	1,242(51%)	492(56%)	410(53%)	340(44%)	
Age (year)^2^					
<=11	1,523(60%)	538(60%)	524(64%)	461(58%)	
>11	985(40%)	356(40%)	292(36%)	337(42%)	
Race or ethnicity (%)^2^					0.22
Mexican American	624(25%)	240(27%)	202(25%)	182(23%)	
Non-Hispanic White	783(31%)	267(30%)	272(33%)	244(31%)	
Non-Hispanic Black	576(23%)	167(17%)	184(23%)	225(28%)	
Other Race	525(21%)	220(25%)	158(19%)	147(18%)	
Family poverty-to-income ratio (%)^2^					0.54
≤1.3	1,083(42%)	394(44%)	349(43%)	340(43%)	
1.3–3.5	865(34%)	309(35%)	279(34%)	277(35%)	
>3.5	560(22%)	191(21%)	188(23%)	181(23%)	
Mother smoked when pregnant (%)^2^					0.87
Yes	302(12%)	111(12%)	103(13%)	88(11%)	
No	2,206(88%)	783(88%)	713(87%)	710(89%)	
Mother’s age when born, year (IQR)^2^	27.0(23.0,31.0)	27.0(23.0,31.0)	28.0(23.0,32.0)	28.0(23.0,31.0)	0.76
Weight at birth, pounds (IQR)^2^	7.00(6.00,8.00)	7.00(6.00,8.00)	7.00(6.00,8.00)	7.00(6.00,8.00)	0.32
Cotinine ng/mL (IQR)^2^	0.03(0.01,0.18)	0.04(0.01,0.25)	0.03(0.01,0.17)	0.03(0.01,0.13)	0.16
BMI kg/m^2^ (IQR)^2^	19.4(16.9,22.9)	19.4(16.7,23.7)	18.9(16.6,22.4)	20.0(17.1,22.9)	0.11
FEV1 (ml/s)^2^	2,367(1,789,3,064)	2,348(1,797,3,087)	2,258(1,725,2,919)	2,518(1,860,3,142)	0.06
FVC (ml)^2^	2,786(2,092,3,518)	2,735(2,097,3,541)	2,662(2,018,3,437)	2,932(2,174,3,633)	0.03

^1^*N* not Missing (unweighted).

^2^*n* (unweighted) (%); Median (Q1, Q3).

^3^Pearson’s X^2: Rao and Scott adjustment; Design-based Kruskal–Wallis test.

^4^Participants were categorized into tertiles (Q1, Q2, and Q3) based on their intake of PUFAs: Q1: <11.48; Q2: 11.48 ≤ Q2 ≤ 16.92; Q3: >16.92.

### 3.2 Association of PUFAs dietary intake with pulmonary function

**Figure 2:**
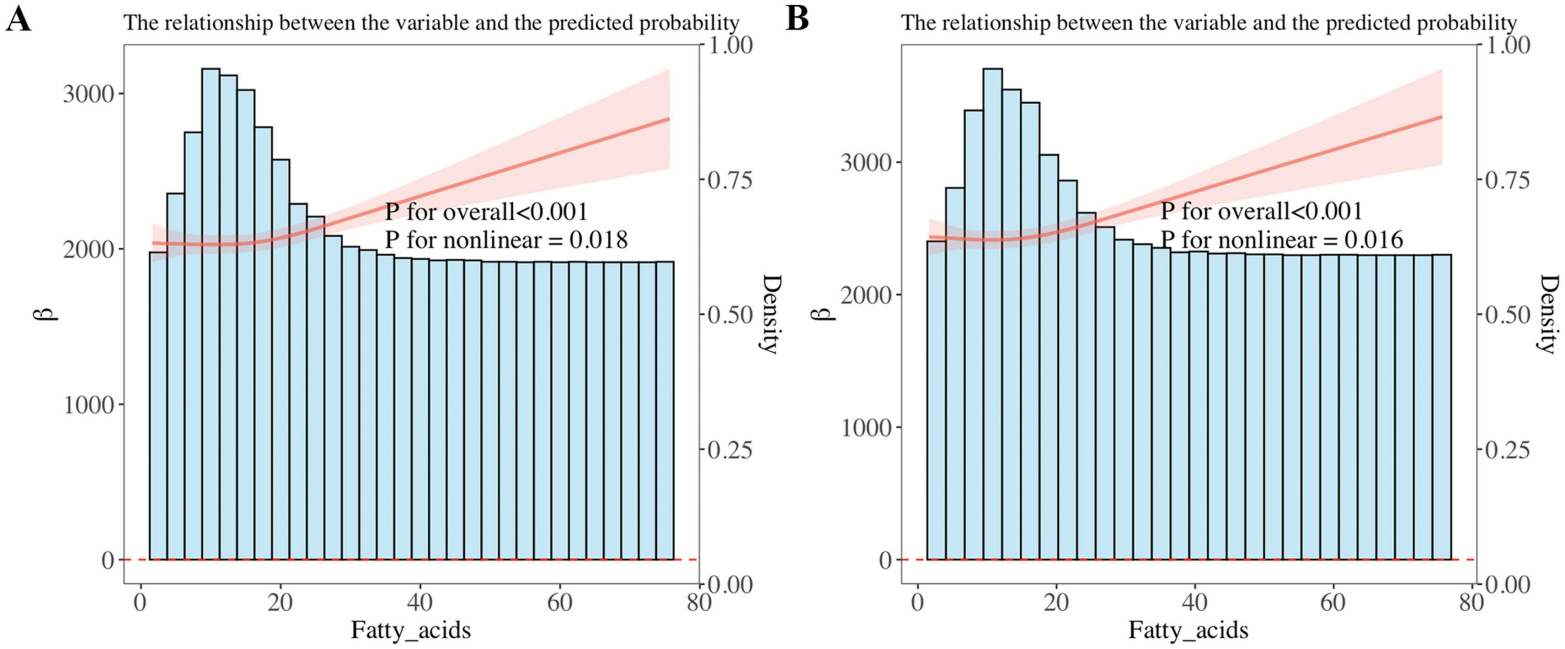
Restricted cubic spline (RCS) analysis showed a non-linear correlation of dietary PUFAs intake with forced expiratory volume in 1 s (FEV1) and forced vital capacity (FVC). FEV1 and FVC increased with increasing PUFA intake. **(A)** FEV1, **(B)** FVC.

Weighted linear regression and RCS were applied to determine the correlation of dietary PUFAs with pulmonary function in children. When the outcome was FEV1, Model 1 was not adjusted for confounders, and PUFAs was positively correlated with pulmonary function in children (*β* = 12.57; 95%CI: 3.919–21.23; *p* = 0.005). After modifying potential confounders gender, age, race and BMI in Model 2, dietary PUFAs intake still showed a positive correlation with pulmonary function in children (*β* = 7.436; 95%CI: 2.827–12.05; *p* = 0.002). Based on Model 2, Model 3 was adjusted for PIR, cotinine, whether the mother smoked during pregnancy, age of mother at birth, child’s birth weight, and it was found that dietary PUFAs was positively correlated with pulmonary function in children (*β* = 7.525; 95%CI: 2.935–12.12; *p* = 0.002). When the outcome was FVC, Model 1 was not adjusted for confounders, and total PUFAs intake was positively correlated with pulmonary function in children (*β* = 15.88; 95%CI: 6.089, 25.66; *p* = 0.002). After modifying potential confounders in Model 2 (*β* = 9.09; 95%CI: 4.321, 13.86; *P* < 0.001) and Model 3 (*β* = 9.138; 95%CI: 4.389, 13.89; *P* < 0.001), PUFAs intake in diet showed a correlation with pulmonary function in children (Table 2). In addition, we further adopted RCS to analyze the linear correlation of PUFAs intake in diet with pulmonary function in children. The results showed that after adjusting for all confounders, there was a non-linear correlation of PUFAs intake in diet with pulmonary function in children (*p* < 0.001) (Figure 2).

**Table 2:** Relationship between polyunsaturated fatty acids (PUFAs) and pulmonary function.

Variables	Model 1		Model 2		Model 3	
	Beta (95%CI)	*p*-value	Beta (95%CI)	*P*-value	Beta (95%CI)	*P*-value
FVC						
Total PUFAs	15.9 (6.1; 25.7)	0.002	9.1(4.3; 13.9)	<0.001	9.1(4.4; 13.9)	<0.001
Total omega-3 PUFAs	149.9(47.2; 252.5)	0.005	96.2(39.9; 152.4)	0.001	95.6(39.2; 152.0)	0.002
Octadecatrienoic	154.2(49.4; 259.1)	0.005	98.7(40.7; 156.7)	0.001	97.9(39.7; 156.1)	0.002
Octadecatetraenoic	3,328(1,0; 5,641)	0.006	793.0(−1,086; 2,672)	0.399	795.6(−995.4; 2,587)	0.374
Eicosapentaenoic	139.3(−1,304; 1,582)	0.847	320.1(−271.7; 911.8)	0.281	300.7(−304.1; 905.4)	0.320
Docosapentaenoic	4,152(−95.4; 8,399)	0.055	1,838(−304.0; 3,979)	0.091	1,961(−137.5; 4,058)	0.066
Docosahexaenoic	91.6(−945.2; 1,128)	0.860	138.9(−319.1; 596.9)	0.544	155.9(−299.8; 611.7)	0.492
Total omega-6 PUFAs	17.1(6.4; 27.8)	0.002	9.7(4.5; 14.9)	<0.001	9.7(4.6; 14.9)	<0.001
Eicosatetraenoic	1,184(233.7; 2,134)	0.016	372.4(−82.0; 826.8)	0.106	391.5(−70.1; 853.1)	0.094
Octadecadienoic	17.1(6.4; 27.9)	0.002	9.7(4.5; 14.9)	<0.001	9.8(4.6; 14.9)	<0.001
Total omega−3/total omega−6	−405.3(−2,724; 1,9,147)	0.727	401.1(−902.1; 1,704)	0.538	401.4(−829.4; 1,632)	0.513
FEV1						
Total PUFAs	12.6(4.0; 21.2)	0.005	7.4(2.8; 12.1)	0.002	7.5(2.9; 12.1)	0.002
Total omega-3 PUFAs	121.1(30.4; 211.8)	0.010	80.5(27.9; 133.0)	0.004	80.6(28.4; 132.8)	0.003
Octadecatrienoic	123.6(30.4; 216.8)	0.010	81.0(26.1; 136.0)	0.005	81.0(26.2; 135.7)	0.005
Octadecatetraenoic	2,818(772.8; 4,864)	0.008	896.6(−738.9; 2,532)	0.275	983.5(−591.8; 2,559)	0.214
Eicosapentaenoic	100.1(−1,101; 1,301)	0.868	300.1(−219.2; 819.4)	0.25	281.0(−204.1; 766.1)	0.248
Docosapentaenoic	3,857(230.5; 7,483)	0.038	2,026(95.1; 3,958)	0.04	2,045(188.1; 3,902)	0.032
Docosahexaenoic	144.6(−758.7; 1,048)	0.749	212.3(−193.3; 617.8)	0.297	224.5(−154.1; 603.0)	0.237
Total omega-6 PUFAs	13.5(4.0; 23.0)	0.006	7.9(2.9; 12.9)	0.003	8.0(3.0; 13.0)	0.003
Eicosatetraenoic	1,068(233.1; 1,903)	0.013	386.8(−16.4; 790.0)	0.06	393.2(−9.1; 795.6)	0.055
Octadecadienoic	13.5(4.0; 23.0)	0.006	7.9(2.9; 13.0)	0.003	8.0(3.0; 13.0)	0.003
Total omega−3/total omega−6	−293.3(−2,382; 1,796)	0.779	366.1(−841.9; 1.6)	0.544	374.6(−774.1; 1.5)	0.513

Model 1: unadjusted.

Model 2: adjusted for Gender, Age, BMI, Race.

Model 3: adjusted for gender, age, BMI, race, PIR, cotinine, whether the mother smoked during pregnancy, age of mother at birth, and child’s birth weight.

### 3.3 The correlation of omega-3 and omega-6 with pulmonary function

**Figure 3:**
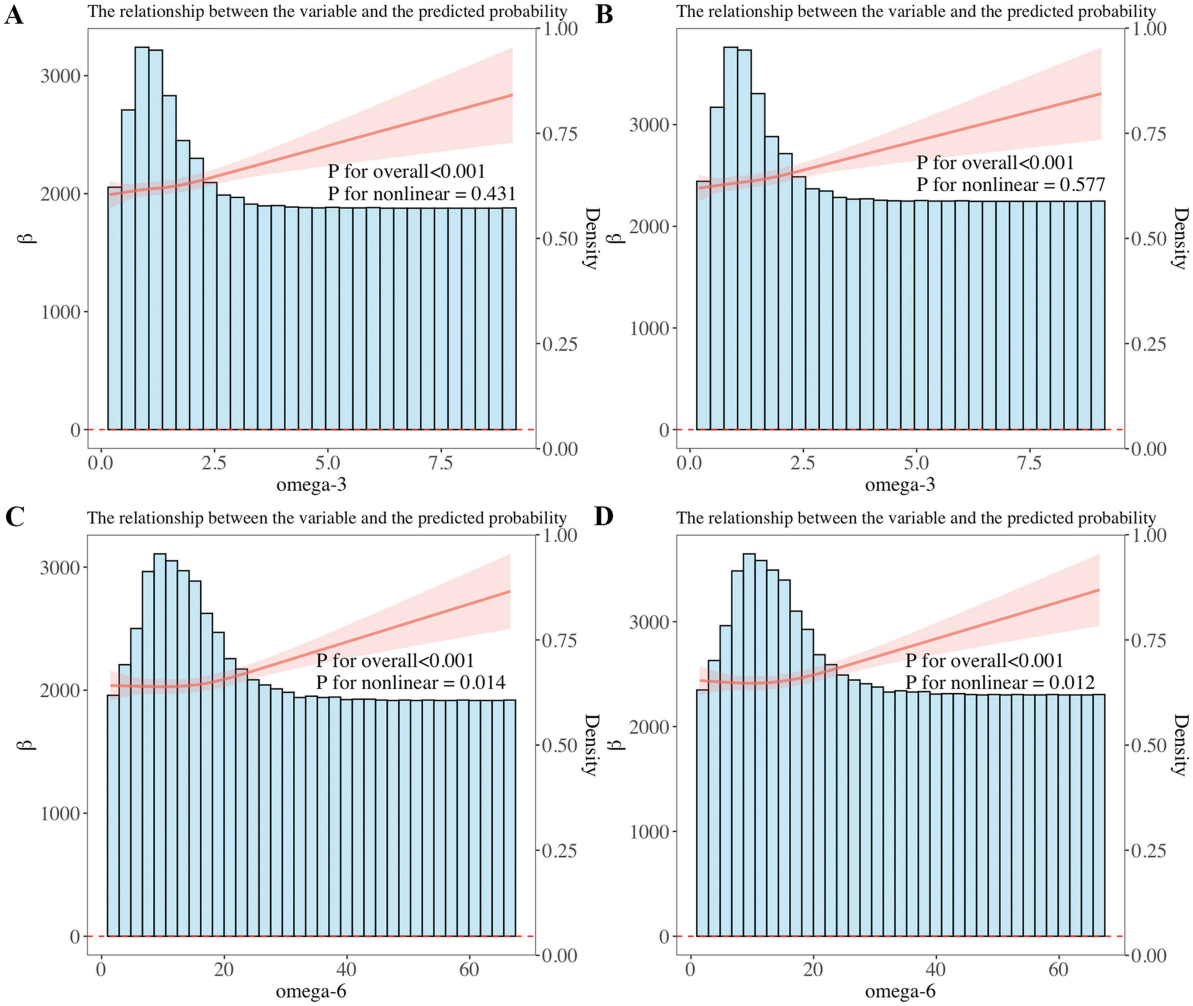
**(A,B)** Restricted cubic spline (RCS) analysis showed a linear relationship of dietary omega-3 intake with forced expiratory volume in 1 s (FEV1) and forced vital capacity (FVC). FEV1 and FVC increased with increasing omega-3 intake. **(C,D)** RCS analysis showed a non-linear correlation between dietary omega-6 intake and FEV1, FVC, with FEV1 and FVC increasing as omega-6 intake increases.

Furthermore, weighted linear regression and RCS were applied to explore the relationship of omega-3 and omega-6 in PUFAs with children’s pulmonary function. The linear regression results showed that after adjusting for all confounders, when the result showed FVC, it increased with the increase of total omega-3 PUFAs (*β* = 95.61; 95%CI: 39.18, 152.0; *p* = 0.002) and total omega-6 PUFAs (*β* = 9.736; 95%CI: 4.588, 14.88; *p* < 0.001) intake. When the result showed FEV1, it increased with the increase of total omega-3 PUFAs (*β* = 80.58; 95%CI: 28.39, 132.8; *p* = 0.003) and total omega-6 PUFAs (*β* = 7.997; 95%CI: 2.998, 12.99; *p* = 0.003) intake. In addition, it was found that the correlation of omega-3 and omega-6 intake with FVC and FEV1 also existed in linear regression models 1 and 2 (*p* < 0.01) (Table 2). We further used RCS analysis to investigate the linear correlation of the intake of omega-3 and omega-6 in children’s diets with pulmonary function (Figure 3). The results showed that after adjusting for all confounders, a non-linear correlation (*p* < 0.05) was observed between omega-6 intake and children’s pulmonary function, as shown in Figure 3.

### 3.4 Subgroup analysis of the correlation of PUFAs intake with pulmonary function

**Figure 4:**
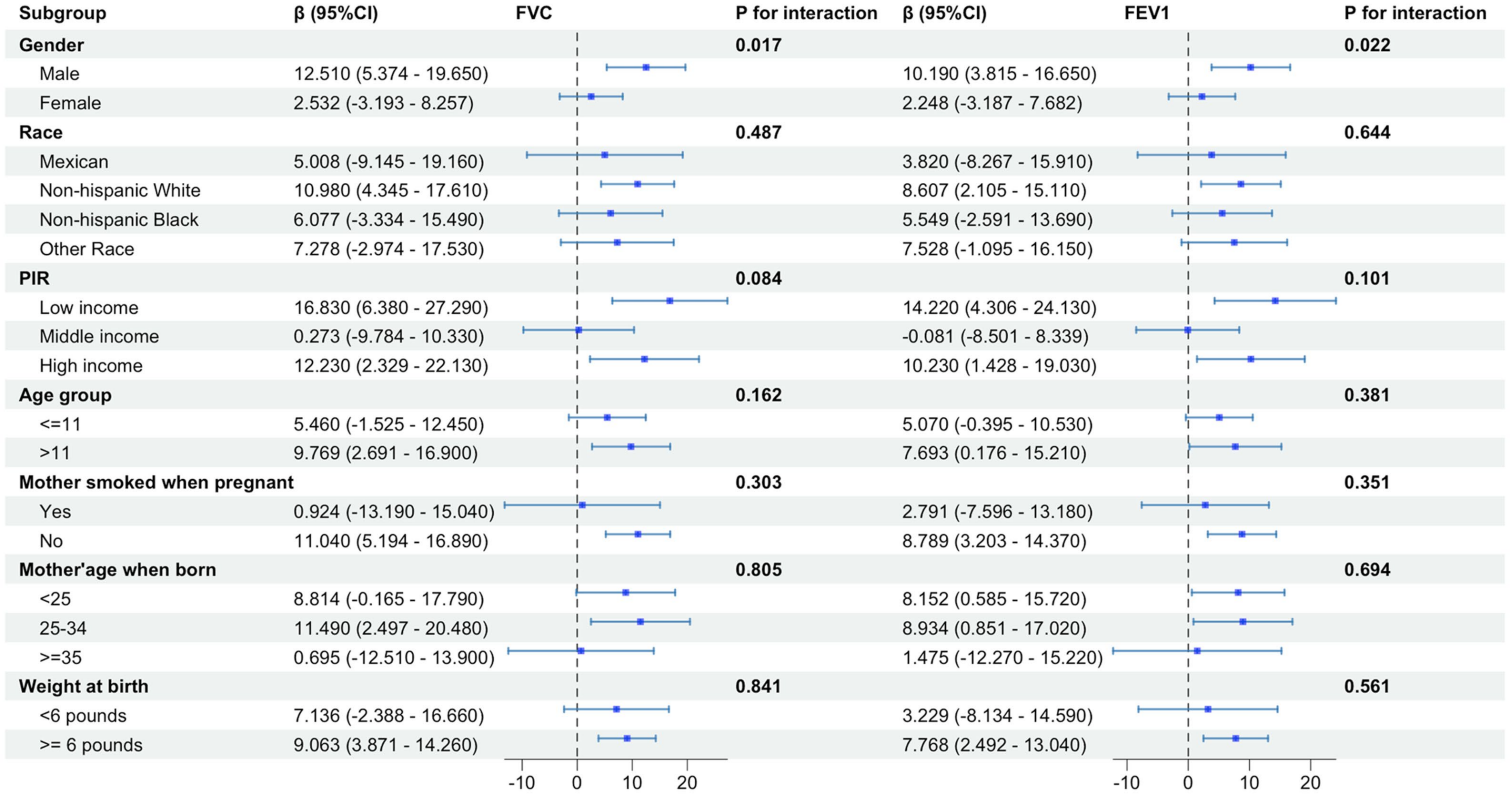
Subgroup analysis of the effect of total PUFAs on forced vital capacity (FVC) and forced expiratory volume in 1 s (FEV1) showed gender differences in the impact of total PUFA intake on pulmonary function, with a more significant impact on pulmonary function in males than in females.

Subgroup analysis was carried out based on gender, race, PIR, age of child, and age of mother at birth to estimate the correlation of PUFAs in diet and pulmonary function in children. Subgroup analysis indicated that dietary PUFAs intake showed an interaction in gender factor (*p* < 0.05) (Figure 4). When the outcome was FEV1, the impact of dietary PUFAs intake on pulmonary function was more significant in males than in females (*β* = 10.190; 95%CI: 3.815–16.650) vs. (*β* = 2.248; 95%CI: −3.187–7.682) (*p* = 0.022). The FVC results showed the same trend with FEV1, with PUFAs having a more significant impact on pulmonary function in males (*β* = 12.510; 95%CI: 5.374–19.650) vs. (*β* = 2.532; 95%CI: −3.193–8.257) (*p* = 0.017). No interaction was found in other subgroup variables.

### 3.5 Subgroup analysis of the correlation of omega-3 and omega-6 intake with pulmonary function

**Figure 5:**
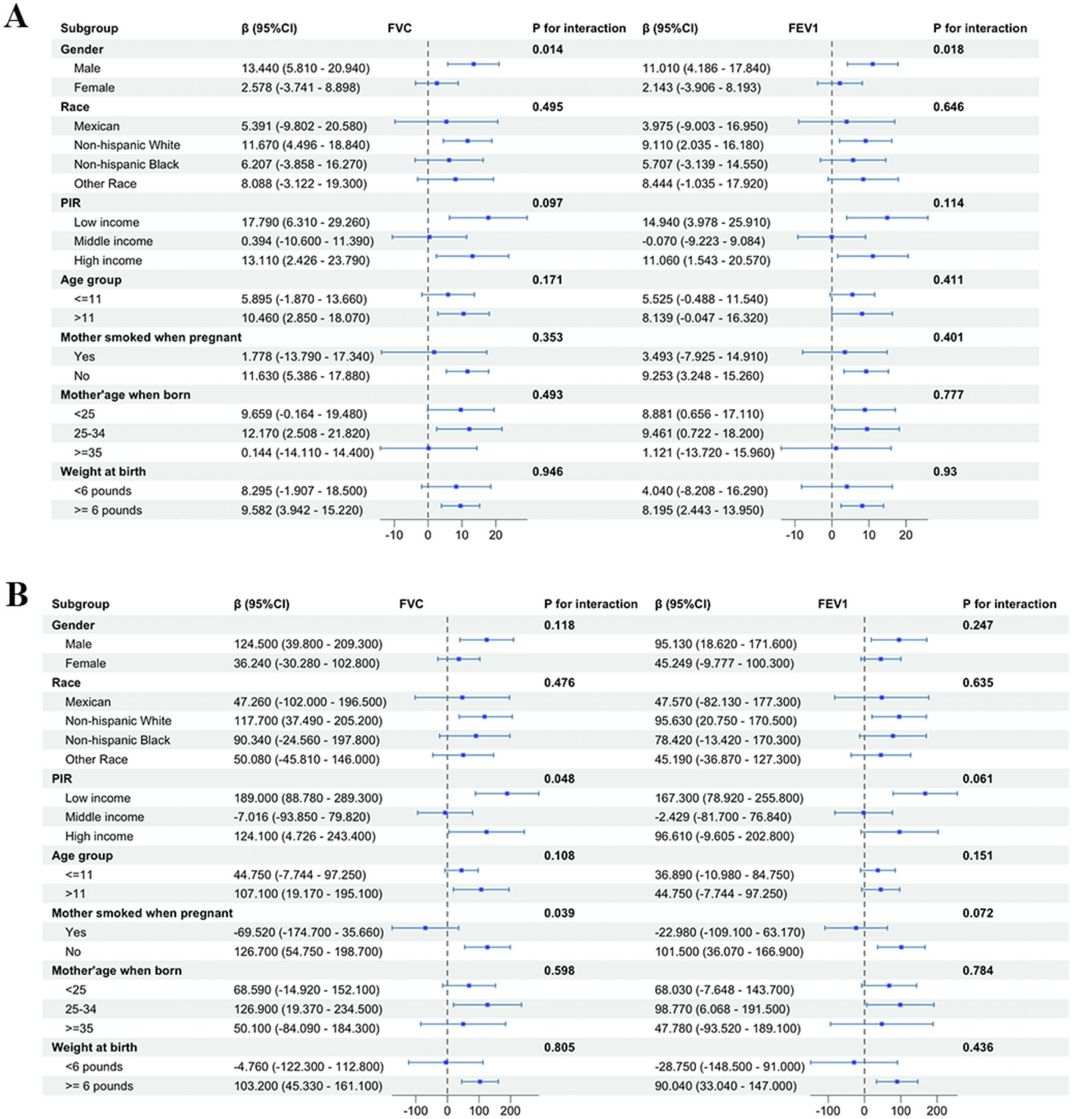
The forest plot of subgroup analysis of forced vital capacity (FVC) and forced expiratory volume in 1 s (FEV1) with omega-6 showed gender differences in the impact of omega-6 on pulmonary function in children in the United States, with a more significant impact on pulmonary function in males than in females. No subgroup differences were found in the subgroup analysis of omega-3. **(A)** Omega-6; **(B)** omega-3.

We further conducted subgroup analysis on the correlation of omega-3 and omega-6 with pulmonary function in children in terms of variables such as gender, race, PIR, age of child, and age of mother at birth (Figure 5). Consistent results with subgroup analysis of total PUFAs were obtained in omega-6, indicating that dietary intake of omega-6 had a more significant impact on pulmonary function in males than in females [FVC: (*β* = 13.440; 95%CI: 5.810–20.940) vs. (*β* = 2.578; 95%CI: −3.741-8.898) (*p* = 0.014); FEV1: (*β* = 11.010; 95%CI: 4.186–17.840) vs. (*β* = 2.143; 95%CI: −3.906–8.193) (*p* = 0.018)].

## 4 Discussion

Our research findings indicate a positive association of the total dietary intake of PUFAs with the pulmonary function of children in the United States. This association was also observed in the subtypes omega-3 and omega-6. In addition, it was also found a gender difference in this correlation, with a more significant correlation in males than in females. To our knowledge, this is the first study to explore the correlation between the intake of PUFAs and pulmonary function in a large and complex cohort of children in the United States.

The correlation of PUFAs with pulmonary function has become a research hotspot in recent years. Previous studies on this correlation mainly focused on the effects of polyunsaturated fatty acid intake on pulmonary diseases, and the research population was mostly adults, with few studies conducted on children. Research on pulmonary diseases mainly involved the relationship of omega-3 with pulmonary inflammation, cancer, and lung infections. Research on the relationship between PUFAs and pulmonary inflammation has found that omega-3 fatty acids can alleviate pulmonary inflammation by inhibiting the release of inflammatory mediators. The specific mechanism involves regulating the expression of cytokines, chemokines, and other inflammatory mediators (37–40). In terms of cancer, recent epidemiological studies have found that high intake of omega-3 fatty acids is associated with a reduced risk of lung cancer, but the specific mechanism still needs further exploration (41–43). In terms of pulmonary infections, some clinical and animal studies have shown that a diet rich in omega-3 fatty acids may enhance the body’s immune function to prevent respiratory infections (27, 44–46). Compared to omega-3, the research on the relationship between omega-6 and pulmonary function is limited and highly controversial. Although omega-6 PUFAs theoretically have pro-inflammatory and pro-thrombotic properties, there is currently no quality data in human studies to support this pro-inflammatory effect, and existing research is highly controversial (14, 47–49). In this context, we selected children in the United States and explored the impact on pulmonary function from three aspects: total PUFAs and their subtypes omega-3 and omega-6.

Our research findings revealed a positive association between the total dietary intake of PUFAs and the pulmonary function of children in the United States. This positive association was also observed in the subtypes omega-3 and omega-6. A non-linear correlation was observed between Omega-6 and pediatric lung function. At low levels of intake, omega-6 did not exhibit a notable impact on enhancing lung function in children. Nevertheless, upon reaching a specific intake threshold, further increases in omega-6 intake were associated with significant improvements in lung function. Although we also assessed the association between PUFA intake and the FEV1/FVC ratio (FEV1%), no significant findings were observed. Thus, we focused on FEV1 and FVC and observed consistent associations, and it was concluded that the two indicators are both well-established measures of lung function. The regulatory mechanisms of the beneficial effects of PUFAs on pulmonary function in previous studies mainly involve the anti-inflammatory and immune regulatory effects of omega-3, as well as the interaction of omega-3 with omega-6. The immunomodulatory effects mainly include the ability of omega-3 to regulate the expression of cytokine genes, modify the membrane fatty acid and phospholipid composition, and displace arachidonic acid (AA, omega-6) in cell membrane phospholipids, thereby reducing inflammation and regulating immune function (13, 50–52). Current research on the interaction between omega-3 and omega-6 suggests that when omega-3 and omega-6 compete for the same metabolic pathway, omega-3 can reduce the production of inflammatory mediators derived from omega-6 (53–55). The impact of omega-6 fatty acids on pediatric pulmonary function is a subject of ongoing debate. For instance, Brigham et al. (27) suggested that the association between omega-6 consumption and asthma symptoms in pediatric populations may be modulated by environmental exposures, specifically air pollution, implying that the observed effects are not exclusively mediated by pro-inflammatory pathways. Additionally, research has indicated that the anti-inflammatory properties of omega-6 are conditional. Under balanced omega-6 to omega-3 ratios, lipid mediators derived from omega-6, such as lipoxins, may exert anti-inflammatory effects by modulating pathways involved in the resolution of inflammation (56). Our findings indicated a positive correlation between omega-6 and pulmonary function in children, potentially reflecting the biological effects of such a balanced state rather than traditionally understood pro-inflammatory pathways. Moreover, our research found a gender difference in the positive relationship between total PUFAs and pulmonary function, which is more significant in males than in females. This finding is consistent with Patchen et al.’s (57) in adult studies. Although studies have reported variations in PUFA metabolism related to sex, race, and ethnicity, McNamara et al. (58) found a sex-related difference in docosahexaenoic acid (DHA) composition in rats, with females exhibiting higher levels than males. Alpha-linolenic acid (ALA) is crucial for maintaining DHA levels in rats, and males are more sensitive to ALA deficiency (58). Ghasemifard et al. (59) reported that sex plays a crucial role in the absorption of fatty acids. Female rats exhibited markedly higher levels of fatty acid absorption than male rats. However, future research still needs to explore the mechanisms of its environment-dependent effects (60).

Overall, the beneficial effects of PUFAs, especially omega-6, on pulmonary function have been highly controversial and uncertain in previous studies, and there is a lack of research on children. This study explored the impact of polyunsaturated fatty acid intake on pulmonary function in a large scale population of children in the United States, providing strong evidence for the future application of PUFAs in the protection of pulmonary function and the prevention of respiratory diseases.

### 4.1 Limitations

There are some limitations in this study. Firstly, the participants of this study are children from the United States, and further confirmation is needed in the future to determine whether the results can be applied to other ethnic groups in other countries. Secondly, the intake of PUFAs in a 24-h diet was determined through recall interviews, which may lead to information bias and measurement errors. Future studies should consider employing more reliable methods such as food diaries or biomarkers. Finally, although efforts were made to adjust for the influencing factors during data analysis, confounding effects of excluded or unknown factors on study findings could not be ruled out.

## 5 Conclusion

The results have shown a positive correlation of the total intake of PUFAs, omega-3 and omega-6, in the diet with pulmonary function in children in the United States, with more obvious correlation found in males. In the future, it is necessary to conduct research on the specific regulatory mechanisms of gender dependence. The study results provide favorable evidence for the protective effect of PUFAs on pulmonary function, and attention should be paid to the intake of PUFAs in children’s daily diet, so as to improve pulmonary function and avoid the occurrence of chronic lung diseases.

## Data Availability

The original contributions presented in the study are included in the article/supplementary material, further inquiries can be directed to the corresponding authors.

## Footnotes

^1^ https://www.cdc.gov/nchs/nhanes/about_nhanes

## References

[1] Schultz ES Hallberg J Andersson N Thacher JD Pershagen G Bellander T. Early life determinants of lung function change from childhood to adolescence. Respir Med. (2018) 139:48–54. doi: 10.1016/j.rmed.2018.04.009, PMID: 29858001

[2] Miller MD Marty MA. Impact of environmental chemicals on lung development. Environ Health Perspect. (2010) 118:1155–64. doi: 10.1289/ehp.0901856, PMID: 20444669 PMC2920089

[3] Belgrave DCM Granell R Turner SW Curtin JA Buchan IE Le Souëf PN. Lung function trajectories from pre-school age to adulthood and their associations with early life factors: a retrospective analysis of three population-based birth cohort studies. Lancet Respir Med. (2018) 6:526–34. doi: 10.1016/s2213-2600(18)30099-7, PMID: 29628377

[4] Agustí A Noell G Brugada J Faner R. Lung function in early adulthood and health in later life: a transgenerational cohort analysis. Lancet Respir Med. (2017) 5:935–45. doi: 10.1016/s2213-2600(17)30434-4, PMID: 29150410

[5] Lange P Celli B Agustí A Boje Jensen G Divo M Faner R. Lung-function trajectories leading to chronic obstructive pulmonary disease. N Engl J Med. (2015) 373:111–22. doi: 10.1056/NEJMoa1411532, PMID: 26154786

[6] Young RP Hopkins R Eaton TE. Forced expiratory volume in one second: not just a lung function test but a marker of premature death from all causes. Eur Respir J. (2007) 30:616–22. doi: 10.1183/09031936.00021707, PMID: 17906084

[7] Talaminos Barroso A Márquez Martín E Roa Romero LM Ortega RF. Factors affecting lung function: a review of the literature. Arch Bronconeumol. (2018) 54:327–32. doi: 10.1016/j.arbres.2018.01.030, PMID: 29496283

[8] Whyand T Hurst JR Beckles M Caplin ME. Pollution and respiratory disease: can diet or supplements help? A review. Respir Res. (2018) 19:79. doi: 10.1186/s12931-018-0785-0, PMID: 29716592 PMC5930792

[9] Shah AM Yang W Mohamed H Zhang Y Song Y. Microbes: a hidden treasure of polyunsaturated fatty acids. Front Nutr. (2022) 9:827837. doi: 10.3389/fnut.2022.827837, PMID: 35369055 PMC8968027

[10] Das UN. Ageing: is there a role for arachidonic acid and other bioactive lipids? A review. J Adv Res. (2018) 11:67–79. doi: 10.1016/j.jare.2018.02.004, PMID: 30034877 PMC6052661

[11] Burdge GC. Metabolism of alpha-linolenic acid in humans. Prostaglandins Leukot Essent Fatty Acids. (2006) 75:161–8. doi: 10.1016/j.plefa.2006.05.013, PMID: 16828546

[12] Calder PC. Omega-3 polyunsaturated fatty acids and inflammatory processes: nutrition or pharmacology? Br J Clin Pharmacol. (2013) 75:645–62. doi: 10.1111/j.1365-2125.2012.04374.x, PMID: 22765297 PMC3575932

[13] Gutiérrez S Svahn SL Johansson ME. Effects of omega-3 fatty acids on immune cells. Int J Mol Sci. (2019) 20:5028. doi: 10.3390/ijms20205028, PMID: 31614433 PMC6834330

[14] Simopoulos AP. The importance of the omega-6/omega-3 fatty acid ratio in cardiovascular disease and other chronic diseases. Exp Biol Med. (2008) 233:674–88. doi: 10.3181/0711-mr-311, PMID: 18408140

[15] Rodríguez-Cruz M Serna DS. Nutrigenomics of ω-3 fatty acids: regulators of the master transcription factors. Nutrition. (2017) 41:90–6. doi: 10.1016/j.nut.2017.04.012, PMID: 28760435

[16] Serhan CN Petasis NA. Resolvins and protectins in inflammation resolution. Chem Rev. (2011) 111:5922–43. doi: 10.1021/cr100396c, PMID: 21766791 PMC3192290

[17] Wang X Li S Wu Y Huang D Pei C Wang Y. Effect of omega-3 fatty acids on th1/th2 polarization in individuals with high exposure to particulate matter ≤ 2.5 μm (pm2.5): a randomized, double-blind, placebo-controlled clinical study. Trials. (2022) 23:1–9. doi: 10.1186/s13063-022-06091-5, PMID: 35209939 PMC8867632

[18] Tong H Zhang S Shen W Chen H Salazar C Schneider A. Lung function and short-term ambient air pollution exposure: differential impacts of omega-3 and omega-6 fatty acids. Ann Am Thorac Soc. (2022) 19:583–93. doi: 10.1513/AnnalsATS.202107-767OC, PMID: 34797737

[19] Yu H Su X Lei T Zhang C Zhang M Wang Y. Effect of omega-3 fatty acids on chronic obstructive pulmonary disease: a systematic review and meta-analysis of randomized controlled trials. Int J Chron Obstruct Pulmon Dis. (2021) 16:2677–86. doi: 10.2147/copd.S331154, PMID: 34588776 PMC8476109

[20] Singer P Bendavid I Mesilati-Stahy R Green P Rigler M Lev S. Enteral and supplemental parenteral nutrition enriched with omega-3 polyunsaturated fatty acids in intensive care patients – a randomized, controlled, double-blind clinical trial. Clin Nutr. (2021) 40:2544–54. doi: 10.1016/j.clnu.2021.03.034, PMID: 33932802

[21] Kim JS Thomashow MA Yip NH Burkart KM Lo Cascio CM Shimbo D. Randomization to omega-3 fatty acid supplementation and endothelial function in copd: the cod-fish randomized controlled trial. Chronic Obstr Pulm Dis. (2021) 8:41–53. doi: 10.15326/jcopdf.8.1.2020.0132, PMID: 33150779 PMC8047614

[22] Choi H Kim T. Polyunsaturated fatty acids, lung function, and health-related quality of life in patients with chronic obstructive pulmonary disease. Yeungnam Univ J Med. (2020) 37:194–201. doi: 10.12701/yujm.2020.00052, PMID: 32252126 PMC7384910

[23] Gutiérrez-Delgado RI Barraza-Villarreal A Escamilla-Núñez C Hernández-Cadena L Garcia-Feregrino R Shackleton C. Effect of omega-3 fatty acids supplementation during pregnancy on lung function in preschoolers: a clinical trial. J Asthma. (2019) 56:296–302. doi: 10.1080/02770903.2018.1452934, PMID: 29617210

[24] Shei RJ Adamic EM Chapman RF Mickleborough TD. The effects of pcso-524®, a patented marine oil lipid derived from the New Zealand green lipped mussel (perna canaliculus), on pulmonary and respiratory muscle function in non-asthmatic elite runners. Int J Exerc Sci. (2018) 11:669–80. doi: 10.70252/HDSA3416 PMID: 29997731 PMC6033491

[25] Bolte G Kompauer I Fobker M Cullen P Keil U Mutius E. Fatty acids in serum cholesteryl esters in relation to asthma and lung function in children. Clin Exp Allergy. (2006) 36:293–302. doi: 10.1111/j.1365-2222.2006.02441.x, PMID: 16499639

[26] Kim HK Kang EY Go GW. Recent insights into dietary ω-6 fatty acid health implications using a systematic review. Food Sci Biotechnol. (2022) 31:1365–76. doi: 10.1007/s10068-022-01152-6, PMID: 36060573 PMC9433510

[27] Brigham EP Woo H McCormack M Rice J Koehler K Vulcain T. Omega-3 and omega-6 intake modifies asthma severity and response to indoor air pollution in children. Am J Respir Crit Care Med. (2019) 199:1478–86. doi: 10.1164/rccm.201808-1474OC, PMID: 30922077 PMC6580674

[28] Hankinson JL Odencrantz JR Fedan KB. Spirometric reference values from a sample of the general u.S. population. Am J Respir Crit Care Med. (1999) 159:179–87. doi: 10.1164/ajrccm.159.1.9712108, PMID: 9872837

[29] Zhang X Qu Y Du L Chen L Luan H Zhou H. Association between omega-6 fatty acid intake and asthma in us children and adolescents. BMC Pediatr. (2024) 24:691. doi: 10.1186/s12887-024-05177-0, PMID: 39478523 PMC11523806

[30] Zhang Z Fulgoni VL Kris-Etherton PM Mitmesser SH. Dietary intakes of epa and dha omega-3 fatty acids among us childbearing-age and pregnant women: an analysis of nhanes 2001-2014. Nutrients. (2018) 10:416. doi: 10.3390/nu10040416, PMID: 29597261 PMC5946201

[31] Nordgren TM Lyden E Anderson-Berry A Hanson C. Omega-3 fatty acid intake of pregnant women and women of childbearing age in the United States: potential for deficiency? Nutrients. (2017) 9:197. doi: 10.3390/nu9030197, PMID: 28245632 PMC5372860

[32] Panjwani AA Cowan AE Jun S Bailey RL. Trends in nutrient- and non-nutrient-containing dietary supplement use among us children from 1999 to 2016. J Pediatr. (2021) 231:131–40.e2. doi: 10.1016/j.jpeds.2020.12.021, PMID: 33340548 PMC8005463

[33] Thompson M Hein N Hanson C Smith LM Anderson-Berry A Richter CK. Omega-3 fatty acid intake by age, gender, and pregnancy status in the United States: national health and nutrition examination survey 2003–2014. Nutrients. (2019) 11:177. doi: 10.3390/nu11010177, PMID: 30650613 PMC6356780

[34] Wang R Feng Y Chen J Chen Y Ma F. Association between polyunsaturated fatty acid intake and infertility among american women aged 20-44 years. Front Public Health. (2022) 10:938343. doi: 10.3389/fpubh.2022.938343, PMID: 36062133 PMC9428268

[35] Skilton MR Raitakari OT Celermajer DS. High intake of dietary long-chain ω-3 fatty acids is associated with lower blood pressure in children born with low birth weight: Nhanes 2003-2008. Hypertension. (2013) 61:972–6. doi: 10.1161/hypertensionaha.111.01030, PMID: 23460284

[36] Wang C Wang L Ding W Zhao F Hou G. Effect of polyunsaturated fatty acids intake on the occurrence of current asthma among children and adolescents exposed to tobacco smoke: Nhanes 2007-2018. J Health Popul Nutr. (2024) 43:168. doi: 10.1186/s41043-024-00663-8, PMID: 39449095 PMC11515328

[37] Rajasinghe LD Chauhan PS Wierenga KA Evered AO Harris SN Bates MA. Omega-3 docosahexaenoic acid (dha) impedes silica-induced macrophage corpse accumulation by attenuating cell death and potentiating efferocytosis. Front Immunol. (2020) 11:2179. doi: 10.3389/fimmu.2020.02179, PMID: 33123123 PMC7573148

[38] He GZ Dong LG Cui XY Chen XF Zhang R. Impact of glutamine and ω-3 polyunsaturated fatty acids on intestinal permeability and lung cell apoptosis during intestinal ischemia-reperfusion injury in a rat model. Zhonghua Wei Chang Wai Ke Za Zhi. (2012) 15:484–9. doi: 10.3760/cma.j.issn.1671-0274.2012.05.021 PMID: 22720351

[39] He GZ Dong LG Chen XF Zhou KG Shu H. Lymph duct ligation during ischemia/reperfusion prevents pulmonary dysfunction in a rat model with ω-3 polyunsaturated fatty acid and glutamine. Nutrition. (2011) 27:604–14. doi: 10.1016/j.nut.2010.06.003 20817408

[40] Bates MA Akbari P Gilley KN Wagner JG Li N Kopec AK. Dietary docosahexaenoic acid prevents silica-induced development of pulmonary ectopic germinal centers and glomerulonephritis in the lupus-prone nzbwf1 mouse. Front Immunol. (2018) 9:2002. doi: 10.3389/fimmu.2018.02002, PMID: 30258439 PMC6143671

[41] Haycock PC Borges MC Burrows K Lemaitre RN Burgess S Khankari NK. The association between genetically elevated polyunsaturated fatty acids and risk of cancer. EBioMedicine. (2023) 91:104510. doi: 10.1016/j.ebiom.2023.104510, PMID: 37086649 PMC10148095

[42] Chen T Song L Zhong X Zhu Q Huo J Chen J. Dietary polyunsaturated fatty acids intake, air pollution, and the risk of lung cancer: a prospective study in Uk biobank. Sci Total Environ. (2023) 882:163552. doi: 10.1016/j.scitotenv.2023.163552, PMID: 37094679

[43] Vega OM Abkenari S Tong Z Tedman A Huerta-Yepez S. Omega-3 polyunsaturated fatty acids and lung cancer: nutrition or pharmacology? Nutr Cancer. (2021) 73:541–61. doi: 10.1080/01635581.2020.1761408, PMID: 32393071

[44] Kim JS Steffen BT Podolanczuk AJ Kawut SM Noth I Raghu G. Associations of ω-3 fatty acids with interstitial lung disease and lung imaging abnormalities among adults. Am J Epidemiol. (2021) 190:95–108. doi: 10.1093/aje/kwaa168, PMID: 32803215 PMC7784523

[45] Bai H Liu T Wang S Wang Z. Polyunsaturated fatty acids, vitamin e and lycopene alleviate ambient particulate matter organic extracts-induced oxidative stress in canine lung cells via the nrf2/ho-1 pathway. Vet Res Commun. (2023) 47:791–801. doi: 10.1007/s11259-022-10040-7, PMID: 36456856

[46] Wiest EF Walsh-Wilcox MT Walker MK. Omega-3 polyunsaturated fatty acids protect against cigarette smoke-induced oxidative stress and vascular dysfunction. Toxicol Sci. (2017) 156:300–10. doi: 10.1093/toxsci/kfw255, PMID: 28115642 PMC6075584

[47] Simopoulos AP. Evolutionary aspects of diet, the omega-6/omega-3 ratio and genetic variation: nutritional implications for chronic diseases. Biomed Pharmacother. (2006) 60:502–7. doi: 10.1016/j.biopha.2006.07.080, PMID: 17045449

[48] Bazinet RP Chu MW. Omega-6 polyunsaturated fatty acids: is a broad cholesterol-lowering health claim appropriate? CMAJ. (2014) 186:434–9. doi: 10.1503/cmaj.130253, PMID: 24218530 PMC3971029

[49] Zhuang P Wang W Wang J Zhang Y Jiao J. Polyunsaturated fatty acids intake, omega-6/omega-3 ratio and mortality: findings from two independent nationwide cohorts. Clin Nutr. (2019) 38:848–55. doi: 10.1016/j.clnu.2018.02.019, PMID: 29551407

[50] Valentine RC Valentine DL. Omega-3 fatty acids in cellular membranes: a unified concept. Prog Lipid Res. (2004) 43:383–402. doi: 10.1016/j.plipres.2004.05.004, PMID: 15458813

[51] Ibarguren M López DJ Escribá PV. The effect of natural and synthetic fatty acids on membrane structure, microdomain organization, cellular functions and human health. Biochim Biophys Acta. (2014) 1838:1518–28. doi: 10.1016/j.bbamem.2013.12.021, PMID: 24388951

[52] Guixà-González R Javanainen M Gómez-Soler M Cordobilla B Domingo JC Sanz F. Membrane omega-3 fatty acids modulate the oligomerisation kinetics of adenosine a2a and dopamine d2 receptors. Sci Rep. (2016) 6:19839. doi: 10.1038/srep19839, PMID: 26796668 PMC4726318

[53] Innes JK Calder PC. Omega-6 fatty acids and inflammation. Prostaglandins Leukot Essent Fatty Acids. (2018) 132:41–8. doi: 10.1016/j.plefa.2018.03.004 29610056

[54] Lorente-Cebrián S Costa AG Navas-Carretero S Zabala M Laiglesia LM Martínez JA. An update on the role of omega-3 fatty acids on inflammatory and degenerative diseases. J Physiol Biochem. (2015) 71:341–9. doi: 10.1007/s13105-015-0395-y, PMID: 25752887

[55] Ekström S Sdona E Klevebro S Hallberg J Georgelis A Kull I. Dietary intake and plasma concentrations of pufas in childhood and adolescence in relation to asthma and lung function up to adulthood. Am J Clin Nutr. (2022) 115:886–96. doi: 10.1093/ajcn/nqab427, PMID: 34964829 PMC8895221

[56] Balić A Vlašić D Žužul K Marinović B Bukvić MZ. Omega-3 versus omega-6 polyunsaturated fatty acids in the prevention and treatment of inflammatory skin diseases. Int J Mol Sci. (2020) 21:741. doi: 10.3390/ijms21030741, PMID: 31979308 PMC7037798

[57] Patchen BK Balte P Bartz TM Barr RG Fornage M Graff M. Investigating associations of omega-3 fatty acids, lung function decline, and airway obstruction. Am J Respir Crit Care Med. (2023) 208:846–57. doi: 10.1164/rccm.202301-0074OC, PMID: 37470492 PMC12042777

[58] McNamara RK Able J Jandacek R Rider T Tso P. Gender differences in rat erythrocyte and brain docosahexaenoic acid composition: role of ovarian hormones and dietary omega-3 fatty acid composition. Psychoneuroendocrinology. (2009) 34:532–9. doi: 10.1016/j.psyneuen.2008.10.013, PMID: 19046819 PMC2692269

[59] Ghasemifard S Hermon K Turchini GM Sinclair AJ. Metabolic fate (absorption, β-oxidation and deposition) of long-chain n-3 fatty acids is affected by sex and by the oil source (krill oil or fish oil) in the rat. Br J Nutr. (2015) 114:684–92. doi: 10.1017/s0007114515002457, PMID: 26234617

[60] Phang M Sinclair AJ Lincz LF Garg ML. Gender-specific inhibition of platelet aggregation following omega-3 fatty acid supplementation. Nutr Metab Cardiovasc Dis. (2012) 22:109–14. doi: 10.1016/j.numecd.2010.04.012, PMID: 20708391

